# Emergence of Extended spectrum β-lactamase (ESBL) and carbapenemase producing *Escherichia coli (E. coli)* in mid-stream urine cultures of patients presenting in outpatient department of tertiary care hospital with uncomplicated cystitis (2016-2022): A retrospective analysis of Laboratory data

**DOI:** 10.12669/pjms.41.1.3379

**Published:** 2025-01

**Authors:** Yusra Shafquat, Riyasat Ahmed Memon, Zahida Shaikh, Ikram Din Ujjan

**Affiliations:** 1 Yusra Shafquat, FCPS Department of Pathology, Liaquat University of Medical and Health Sciences, Jamshoro, Pakistan; 2 Riyasat Ahmed Memon, FCPS Department of Pathology, Bilawal Medical College, Liaquat University of Medical and Health Sciences, Jamshoro, Pakistan; 3 Zahida Shaikh, MPhil Department of Pathology, Liaquat University of Medical and Health Sciences, Jamshoro, Pakistan; 4 Ikram Din Ujjan, PhD Department of Pathology, Liaquat University of Medical and Health Sciences, Jamshoro, Pakistan

**Keywords:** Uncomplicated cystitis, *E. coli*, Antimicrobial resistance, Urinary tract Infection, empirical therapy

## Abstract

**Objective:**

To determine the prevalence of antimicrobial resistance (AMR) in *E. coli* isolated from urine cultures of patients with uncomplicated cystitis in Pakistan. Another objective was to analyze and compare the resistance rates of *E. coli* to specific antibiotics, conducting a year-by-year evaluation of these rates to identify trends and changes over the past seven years.

**Methods:**

Retrospective analysis of susceptibility data of *E. coli* isolated from midstream urine culture samples of patients presenting in outpatient department with uncomplicated cystitis, from January 2016 to December 2022 in the section of Microbiology, Liaquat University of Medical and Health Sciences was done. All the demographic data, clinical information and susceptibility results were obtained from laboratory data base. All the cultures were performed on CLED agar and *E. coli* was identified using biochemical tests, susceptibility test was performed by disk diffusion method and clavulanate inhibition test for analysis of extended spectrum β-lactamase (ESBL) was performed.

**Results:**

A total of 5169 patients with uncomplicated cystitis with no history of renal disease, pregnancy, hospitalization, catheterization and no elderly or pediatric population were included, 76% of which were of females. Mean age was 37 ±11 years Rising trends of resistance were observed in ampicillin (86.3%-95.3%), cotrimoxazole (12.8-48.6%), ciprofloxacin (57.3-81.6%) and low rates of resistance were seen in fosfomycin (0.57-2.96%), nitrofurantoin (0.72-2.96%) and amikacin (2.09-3.15%). Presence of ESBL and emerging resistance to ceftriaxone (15.7-48.7%), piperacillin/tazobactam (0-4.84%) and carbapenems (0-0.39%) was observed.

**Conclusion:**

The national guidelines offer an empirical treatment regimen for patients with uncomplicated cystitis. However, regional variations in antimicrobial resistance (AMR) complicate adherence to these guidelines. Our study supports the use of fosfomycin and nitrofurantoin in management of uncomplicated cystitis, However, ongoing annual regional surveillance is essential to keep clinicians informed about the latest trends in AMR.

## INTRODUCTION

Uncomplicated cystitis is a common infection, which is known to affect more than 1.2 million people annually.^1^ The risk factors such as old age, prior history of urinary tract infection (UTI), frequent intercourse, diabetes mellitus and low socioeconomic factors, have led to an increase in the incidence of uncomplicated cystitis and recurrent UTI.^2^ Community acquired uncomplicated infection is mostly caused by *Escherichia coli (E. coli) having a prevalence range of 70-90%*.^3^ A systemic review over a decade regarding AMR in Pakistan showed UTI as the most reported diagnosis and with resistance to multiple drugs in urinary tract infections along with other systemic infections of our body.^4^ According to the Guidelines for antimicrobial use by the infectious diseases society of Pakistan, culture is not recommended for the diagnosis of acute uncomplicated cystitis and the recommended empirical regimen includes ciprofloxacin, amoxicillin/clavulanic acid, cefixime and fosfomycin. It also states that the pathogen is less likely to be an ESBL producing *E. coli*.^5^

Asia is known to have rising antimicrobial resistance, the rate being as high as 70%, with Pakistan ranked fifth amongst the countries reporting rising trends of antimicrobial resistance.^6^ This increasing rate is a major public threat for the people of Pakistan and may spread by travel- tourists and immigrants.^6^ This increasing resistance is mainly due to injudicious use of antibiotics in humans and animals both.^6^

The rising rates of antimicrobial resistance (AMR) among uropathogenic *E. coli* strains pose a significant public health threat, particularly in regions like Pakistan, where empirical treatment guidelines may not align with local resistance patterns. Given that uncomplicated cystitis is prevalent among the population, understanding the local AMR landscape is crucial for effective management. This study aimed to provide updated and region-specific data on AMR trends, including the prevalence of extended-spectrum beta-lactamase (ESBL) and carbapenemase-producing strains. By identifying the resistance patterns and associated risk factors, this research will equip clinicians with the necessary information to make informed decisions on empirical therapy, reduce the irrational use of antibiotics, and contribute to antibiotic stewardship efforts in Pakistan. Such data are essential not only for optimizing patient care but also for mitigating the broader implications of AMR in the community.

## METHODS

This retrospective study was carried out at the Microbiology Section of the Diagnostic and Research Laboratory at Liaquat University of Medical and Health Sciences in Hyderabad, Pakistan. Using a consecutive sampling technique, we included all mid-stream urine samples collected from patients aged 18 to 60 years who presented to the outpatient department of Liaquat University Hospital with uncomplicated cystitis between January 2016 and December 2022. Liaquat University Hospital is a tertiary care facility with 1,450 beds, serving one of the largest outpatient departments in the region, attracting patients from both urban and rural areas of Sindh, Pakistan.

### Ethical Approval:

This study was exempted from ethical approval by the Institutional Ethical Review Committee (LUMHS/PATH/BMS/-30/6167- dated: April 01, 2019)

### Study Participants:

**Fig.1:**
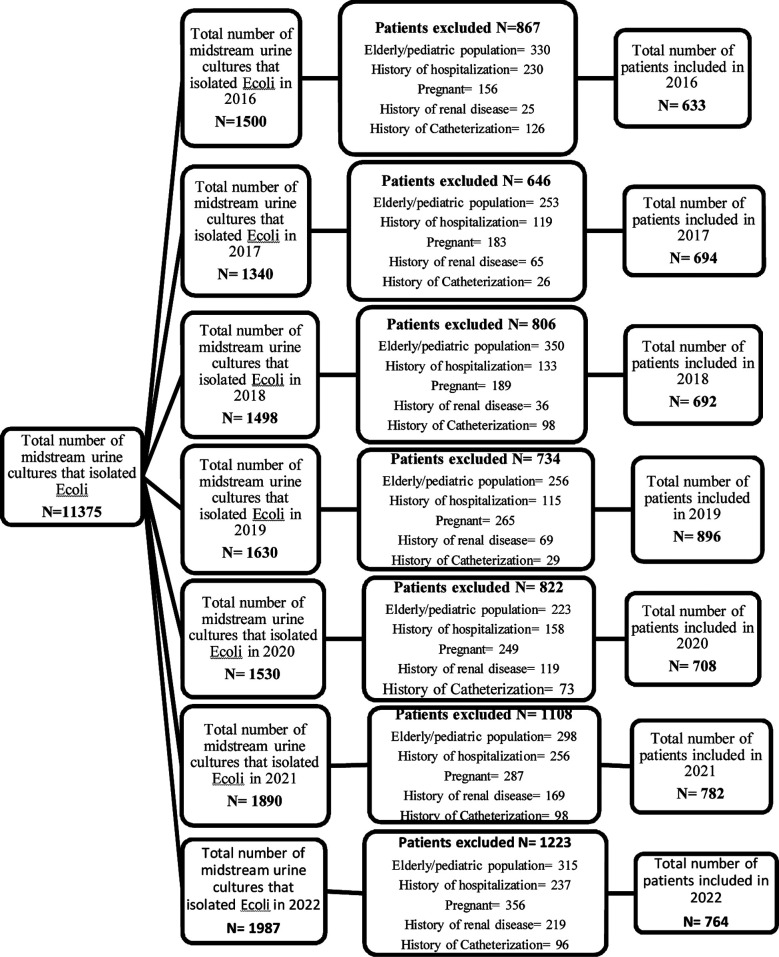
Flowchart showing study design and selection of participants.

Patients presenting with dysuria, frequency, urgency, flank pain and burning micturition were diagnosed as having uncomplicated cystitis were included. Patients who had a history of hospitalization, pregnancy, renal disease, structural abnormality and catheterization were excluded (Fig.1). All the data was retrieved from the centralized data system (LIMS) and included: Identification, age, gender, location (outpatient or inpatient), year and month of isolation, brief clinical history, organism isolated and antimicrobial susceptibility pattern. The data obtained were entered into statistical software SPSS version 19.0 (SPSS Inc., Chicago, IL).

### Microbiological Methods:

All clinical midstream urine samples were inoculated on cysteine lactose electrolyte deficient (CLED) agar and incubated aerobically at 37ºC for 24 and 48 hours. Culture with the growth of uropathogen in the count of >10^5^ was considered significant. *E. coli* was identified using biochemical methods, which included indole, motility and triple sugar iron (TSI) tests.

### Antimicrobial susceptibility tests:

Antimicrobial susceptibility testing was performed by Kirby Bauer disk diffusion method and interpreted as per Clinical Laboratory Standards Institute (CLSI) on Mueller hinton agar (MHA).^7^ Antimicrobial discs tested were ampicillin (10 µg), amoxicillin-clavulanic acid (20/10 µg), ciprofloxacin (10 µg), fosfomycin (50µg), nitrofurantoin (300 µg), piperacillin-tazobactam (100/10 µg), ceftriaxone (30 µg), gentamicin (10 µg), amikacin (30 µg), trimethoprim-sulfamethoxazole (1.25/23.75 µg), and imipenem (10 µg) (Oxoid™, Thermo scientific™, Basingstoke, Hants, UK).

### Disk Diffusion clavulanate inhibition test for ESBL:

All the strains that had a zone diameter of <25mm of ceftriaxone by disk diffusion method were further tested for ESBL production by clavulanate inhibition test. Ceftazidime (30 µg) and ceftazidime clavulanate (30/10 µg) were tested for these strains on MHA. Increase >5mm in zone diameter of ceftazidime-clavulanate when compared to ceftazidime alone showed the presence of ESBL.

### Statistical Analysis:

The data obtained were entered into statistical software SPSS version 19.0 (SPSS Inc., Chicago, IL). For descriptive analysis, mean and standard deviation of continuous variables such as age were computed. Frequencies and percentages were calculated for categorical variables such as gender and antimicrobial resistance.

## RESULTS

A total of 5169/11375 clinical midstream urine samples that isolated significant count of *E. coli*, of patients who presented in outpatient department with uncomplicated cystitis with no history of renal disease, pregnancy, hospitalization, catheterization and no elderly or pediatric population were included, 76% of which were females. Mean age was 37 ±11 years.

### Antimicrobial susceptibility data:

The rates of resistance of ampicillin (86.3-95.3%), Amoxicilllin-clavulanate (65.7-97.4%) and ciprofloxacin (57.3-81.6%) were high in all the seven years. Fosfomycin (0.57-2.96%) and nitofurantoin (0.72-2.96%) were the only oral options that had low resistance rates (<5%). Emerging resistance to ceftriaxone (15.7-48.7%), piperacillin/tazobactam (0-4.84%) and carbapenems (0-0.39%) was observed, (Table-I).

**Table-I:** Trends of Antimicrobial resistance observed over a period of 7 years (2016-2022).

Year	Amp N (%)	AMC N (%)	AK N (%)	CN N (%)	FOT N (%)	F N (%)	SXT N (%)	CIP N (%)	CRO N (%)	MEM N (%)	TZP N (%)
2016 N= 633	561 (88.6)	441 (69.6)	20 (3.15)	55 (8.68)	25 (3.94)	13 (2.05)	252 (39.8)	363 (57.3)	100 (15.7)	0	0
2017 N= 694	661 (95.2)	456 (65.7)	18 (2.59)	13 (1.87)	4 (0.57)	5 (0.72)	115 (16.57)	445 (64.1)	134 (19.3)	0	0
2018 N= 692	616 (89.0)	600 (97.4)	10 (1.44)	107 (15.4)	8 (1.15)	10 (1.44)	99 (14.3)	565 (81.6)	130 (18.8)	0	4 (0.57)
2019 N= 896	795 (88.7)	793 (88.5)	20 (2.23)	225 (25.1)	9 (1.00)	16 (1.78)	115 (12.8)	663 (73.9)	155 (17.2)	0	15 (1.67)
2020 N= 708	675 (95.3)	616 (87.0)	25 (3.53)	263 (37.1)	15 (2.12)	21 (2.96)	225 (31.7)	559 (78.5)	255 (36.0)	0	21 (2.96)
2021 N= 782	699 (89.4)	669 (85.5)	21 (2.68)	225 (28.7)	8 (1.02)	22 (2.81)	305 (39.0)	601 (76.8)	363 (46.4)	2 (0.25)	25 (3.19)
2022 N= 764	660 (86.3)	696 (91.1)	16 (2.09)	121 (15.8)	20 (2.61)	19 (2.48)	372 (48.6)	592 (77.5)	372 (48.7)	3 (0.39)	37 (4.84)

Amp- Ampicillin, AMC- Amoxicilllin-clavulanate, Ak-Amikacin, CN- Gentamicin, FOT- Fosfomycin,F- Nitrofurantoin, SXT- Cotrimoxazole, CIP- Ciprofloxacin, CRO- Ceftriaxone, MEM- Meropenem, TZP - Piperacillin- tazobactam.

### Presence of ESBL producing E. coli:

**Fig.2:**
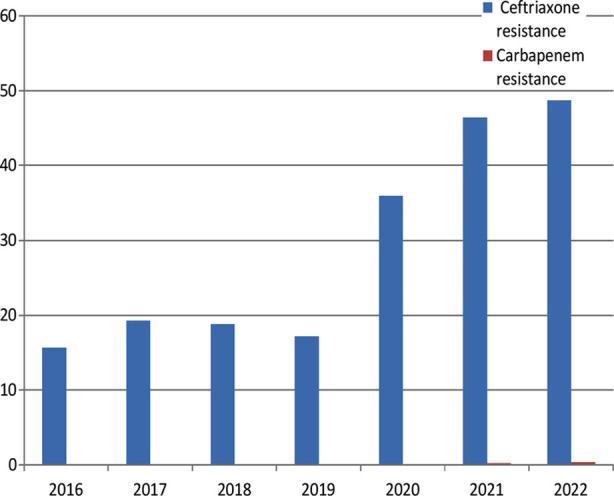
Increasing trend of ceftriaxone and carbapenem resistance over the period of 7 years.

1509 isolates were resistant to ceftriaxone and were subjected to disk diffusion clavulanate inhibition test for presence of ESBL. Interestingly all the strains showed the presence of ESBL (29.1%), with highest number being in 2022 (48.7%) (Fig.2).

### Frequency of Carbapenem resistant E. coli:

There were only 2 (0.25%) and 3 (0.39%) cases of carbapenem resistance observed in 2021 and 2022.

## DISCUSSION

This study reveals a consistently high resistance rate in uropathogenic strains of E. coli to ampicillin, amoxicillin-clavulanate, and ciprofloxacin, along with a notable increase in resistance to cephalosporins and cotrimoxazole. In contrast, resistance to oral antibiotics such as fosfomycin and nitrofurantoin remained below 5%, supporting their continued use as empirical treatments for uncomplicated cystitis. Additionally, the presence of extended-spectrum beta-lactamase (ESBL) in many strains has contributed to the rising resistance against cephalosporins. Notably, we observed an upward trend in resistance to piperacillin/tazobactam and emerging resistance to carbapenems, highlighting significant challenges for empirical antibiotic prescribing.

In a study conducted in Kohat, Pakistan, high rates of resistance to ampicillin, cephalosporins and ciprofloxacin were observed. However, the study also showed 50% resistance to meropenem whereas our study showed an imperceptible rate of resistance to carbapenems.^8^ The possible explanation for this geographical variation in antimicrobial susceptibility pattern is due to difference in empirical prescription of drugs, availability of over the counter drugs and overconsumption.^9^-^11^ Prescription of high dose and shorter regimens, may help reduce the emerging resistance.^11^

Our findings are consistent with a study conducted in Karachi, Pakistan with the difference that our study also highlights the emerging resistance to cephalosporins and carbapenem. Similarly, in an another four month study conducted in outpatient department of a tertiary care hospital in Karachi showed E. coli as the most frequent cause of uncomplicated cystitis and with 62% and 7% resistance to cephalosporins and carbapenem.^12^ In Potohar region, the frequency of ESBL producers is as high as 57% with 94% resistance to 3^rd^ generation cephalosporins and 2% to carbapenem.^13^ In Bangladesh, the resistance to 3^rd^ generation cephalosporins and carbapenems is 50.2% and 2.4% in uropathogenic E. coli, respectively.^14^ The findings of our study are consistent with many studies conducted worldwide, highlighting the now evolving and rapidly spreading resistance, mandating prompt action to contain it.^15^-^18^

The use of ciprofloxacin and cephalosporins in Pakistan has increased by 1.86 DIDs (Defined daily dose/ 1000 inhabitants/ day) as analyzed by a five year study (2014-2018).^19^ Our study shows high rate of resistance to ciprofloxacin. So, it cannot be used for empirical management of community acquired UTI. The inappropriate and irrational use of drugs due to easy accessibility is a global problem and has led to increased treatment costs, complications and emerging resistance. Antibiotic stewardship programs are needed across the country to control the alarming rising rates of resistance.^19^ National Action Plan of Pakistan on antimicrobial resistance, released in 2017, devised by Ministry of Health services, is taking steps to strengthen the health system of Pakistan, so as to implement antibiotic stewardship, thereby reducing antimicrobial resistance.^17^,^20^

A study conducted in India showed 34.4% of community acquired UTI caused by ESBL producing *E. coli* whereas our study shows slightly lower rates, however considering the trends observed higher rates are expected in future^21^. These findings suggest that ESBL detection tests should be performed routinely in all urine cultures. The study under discussion also highlighted a very high resistance rate to nitrofurantoin (80%), which whereas ours was only 2-3%.^21^ These regional variations in antimicrobial susceptibility pattern mandate regular antimicrobial resistance surveillance and update of antibiotic susceptibility data. Also, our data suggests that ESBL producing strains can be a cause of community acquired acute uncomplicated cystitis as opposed to the guidelines and this should be kept in mind while empirical prescription so as to avoid an increase in AMR.^5^

In April 2018, National Action Plan team of Pakistan presented a situation analysis report that highlighted various factors responsible for antimicrobial resistance in Pakistan and which included unnecessary prescription, availability and registration of clinically irrelevant products, deceiving advertisements and quacks and irrational use of antibiotics in animals, poultry and agriculture.^20^ For AMR surveillance, laboratories of resource limited setting need to be provided with support and careful mentoring so as to ensure quality assured diagnostic tools and skilled personnel.^22^ Together all the three key members dealing with AMR- Clinician, laboratory personnel and pharmacists can help contain the evolving problem of AMR.

### Strengths:

The strengths of our study include evaluation of a large number of samples, longer duration of study, evenly distributed study population with comprehensible inclusion and exclusion criteria and a diverse study population representing various urban and rural regions of province Sind, Pakistan. Our study highlights the emerging resistance to cephalosporins and carbapenems (which was previously known to be possible only in hospital acquired infections) in community acquired infections.

### Limitations:

The limitations of our study include retrospective nature of study, no separate data and analysis of recurrent UTI in participants, other uropathogens were not assessed and there was no evaluation of carbapenemase detection and antimicrobial resistance genes.

Despite many limitations, this retrospective single-center study is crucial for several reasons. First, it addresses the urgent need for updated data on antimicrobial resistance (AMR) in uropathogenic *E. coli*, particularly in the context of uncomplicated cystitis, a common infection affecting many patients. By focusing on a specific region in Pakistan, the study provides localized insights that can inform clinical practices and treatment guidelines tailored to the area’s unique resistance patterns. Second, the findings underscore the persistently high rates of resistance to commonly used antibiotics, which can have significant implications for patient management and public health. Understanding these resistance trends is essential for clinicians to make informed decisions about empirical therapy, ultimately leading to more effective treatment and reduced antibiotic misuse.

Additionally, the study highlights the importance of monitoring the emergence of resistance to broad spectrum antibiotics, such as carbapenems, which are critical for treating severe infections. By identifying these trends early, healthcare providers can implement strategies to mitigate the spread of resistance and enhance antibiotic stewardship efforts.

Finally, this research contributes to the broader understanding of AMR in Pakistan and supports the development of national policies aimed at combating this growing public health threat. Overall, the study not only fills a critical gap in the existing literature but also serves as a foundation for future research and interventions to improve patient outcomes and control AMR in the community.

## CONCLUSION

In conclusion, our study reveals that resistance of uropathogenic *E. coli* to ampicillin, amoxicillin-clavulanate, ciprofloxacin, ceftriaxone, and cotrimoxazole exceeds 10%, rendering these antibiotics unsuitable as empirical treatments for uncomplicated urinary tract infections (UTIs). The emergence of resistance to piperacillin/tazobactam and carbapenems is particularly concerning, highlighting the urgent need for effective antibiotic stewardship and ongoing surveillance. Conversely, fosfomycin and nitrofurantoin, in line with national guidelines, can currently be utilized safely as empirical agents for uncomplicated cystitis. This research not only aids in the empirical management of UTIs but also contributes to the enhancement of the national database and the strategic development of antimicrobial resistance education and surveillance initiatives.

### Authors’ Contribution:

**YS:** Conceived, designed and collected, interpreted and did statistical analysis of data & writing and editing of manuscript, is responsible for integrity of research and gave final approval of manuscript.

**RAM:** Collected, interpreted and analysed data, manuscript writing and final approval of manuscript

**ZS:** Analyses data, reviewed, edited and final approval of manuscript.

**IDU:** Reviewed, edited and final approval of manuscript.
